# The Impact of Gestational Diabetes Mellitus on Minipuberty in Girls

**DOI:** 10.3390/ijms252111766

**Published:** 2024-11-01

**Authors:** Karolina Kowalcze, Sofia Burgio, Giuseppe Gullo, Joanna Kula-Gradzik, Johannes Ott, Robert Krysiak

**Affiliations:** 1 Department of Pediatrics in Bytom, Faculty of Health Sciences in Katowice, Medical University of Silesia, Stefana Batorego 15, 41-902 Bytom, Poland; jkula-gradzik@sum.edu.pl; 2 Department of Pathophysiology, Faculty of Medicine, Academy of Silesia, Rolna 43, 40-555 Katowice, Poland; 3 Department of Obstetrics and Gynecology, Villa Sofia Cervello Hospital, University of Palermo, 90146 Palermo, Italy; sofiaburgio4@gmail.com (S.B.); gullogiuseppe@libero.it (G.G.); 4 Clinical Division of Gynecologic Endocrinology and Reproductive Medicine, Department of Obstetrics and Gynecology, Medical University of Vienna, 1090 Vienna, Austria; johannes.ott@meduniwien.ac.at; 5 Department of Internal Medicine and Clinical Pharmacology, Medical University of Silesia, Medyków 18, 40-752 Katowice, Poland; rkrysiak@sum.edu.pl

**Keywords:** female infants, hypothalamic–pituitary–ovarian axis, hyperglycemia, neonatal complications, saliva, sexual organs, urine

## Abstract

Minipuberty is the second phase of physiological activation of the reproductive axis, playing a role in the postnatal development of sexual organs. The course of female minipuberty was found to be affected by low maternal vitamin D status and hypothyroidism during pregnancy. The aim of the current study was to assess the hormonal profile and the size of sexual organs in daughters of mothers with gestational diabetes mellitus. The study included three matched groups of infant girls: daughters of healthy women without metabolic disorders during pregnancy (group 1), daughters of women with poorly controlled gestational diabetes mellitus (group 2), and daughters of women with gestational diabetes mellitus adequately controlled during pregnancy (group 3). Urinary levels of gonadotropins, salivary levels of estradiol, testosterone, DHEA-S and progesterone, ovarian volume, uterine length and breast diameter were measured from postnatal month 1 to postnatal month 18. Concentrations of FSH, LH and estradiol were higher, while concentration of progesterone was lower in group 2 than in the remaining groups. There were no between-group differences in concentrations of testosterone and DHEA-S. Levels of LH, FSH, estradiol and progesterone correlated with maternal whole-blood levels of glycated hemoglobin. Group 2 was also characterized by the longest detection periods for LH and estradiol. Ovarian volume, uterine length and breast diameter were greater in group 1 than in the remaining two groups. Over the entire observation period, there were no differences in hormone levels and sizes of the sexual organs between groups 1 and 3. The obtained results suggest that poorly controlled, but not well controlled, gestational diabetes mellitus affects the course of female minipuberty.

## 1. Introduction

Gestational diabetes mellitus is defined as carbohydrate intolerance causing hyperglycemia of variable severity with onset or first recognition during pregnancy [1]. Affecting up to 15–25% of pregnancies worldwide, it is considered the most prevalent disease among pregnant women [2]. Children born to women with gestational diabetes mellitus are at a greater risk of perinatal mortality, macrosomia, prematurity, birth injury, shoulder dystocia, respiratory distress, neonatal jaundice, admission to neonatal care and type 2 diabetes in later periods of life [3,4]. Identification of gestational diabetes mellitus and appropriate treatment during pregnancy are important to reduce the risk of these adverse outcomes [5].

There are three phases of physiological activation of the hypothalamic–pituitary–gonadal axis: the first one occurs during fetal life, the second during the postnatal and infant period, and the third at puberty [6]. The second phase of a transient, sex-specific surge of luteinizing hormone (LH), follicle-stimulating hormone (FSH) and gonadal steroids is known under the name of minipuberty [7,8]. Female minipuberty is characterized by a biphasic course, with an initial peak in circulating levels of gonadotropins and estradiol at around days 15 to 27 and a less expressed second peak at around weeks 15 to 24 [9]. After minipuberty, the reproductive axis remains in a state of relative quiescence until its reactivation promotes puberty [10]. In comparison to male minipuberty, this phase of activation of the reproductive axis in girls is characterized by higher levels of FSH and estradiol, as well as by their longer detection periods: even until the second year of age for estradiol and even until 3–4 years of age for FSH [7,8]. Elevated gonadotropin and estrogen production in females in the first postnatal life may determine early ovarian, uterine and mammary growth [6]. The sex-specific course of minipuberty may also contribute to sex-related differences in growth velocity and fat distribution [11,12]. Lastly, early activation of the reproductive axis may be implicated in structural and functional brain development, affecting brain plasticity, determining gender identity and playing a role in the development of language and emotional competencies [11,13].

Female offspring of mothers with gestational diabetes mellitus were found to be characterized by an earlier pubertal onset, as well as by a faster speed of pubic hair and breast development [14,15,16,17]. Prenatal exposure to hyperglycemia was also associated with an earlier age at menarche and with very early menarche, defined as the first occurrence of menstruation in girls aged 10 years or younger [16,18]. The association with pubertal timing was particularly evident in daughters of women with gestational diabetes mellitus and pregravid body mass index (BMI) greater than or equal to 25 kg/m^2^ [17]. Although prenatal exposure to gestational hyperglycemia often results in increased adipose tissue content [4], the association between gestational diabetes and pubertal development was found to persist after adjustment for BMI [19]. The impact of maternal gestational diabetes mellitus on puberty was more evident in girls than in boys, in whom earlier pubertal timing was inconsistently observed [14,15,16]. The impact of sex may explain no statistically significant differences in the age of puberty onset when girls and boys were analyzed together [20]. Unfortunately, the studies suggesting earlier timing of pubarche, thelarche and menarche in the offspring of mothers with gestational diabetes mellitus were widely heterogeneous in terms of the investigated populations and outcome measures [21]. Interestingly, the impact of maternal diabetes on puberty differs from the impact of hyperglycemia in the child itself. Diabetes (mainly type 1) was found to be associated with later pubertal development, and the delay was found to correlate with the disease duration and the degree of metabolic control. Pubertal development was, however, either less delayed or even normal in patients achieving therapeutic goals [22,23,24].

The course of minipuberty is altered in female descendants of women with chronic health problems during pregnancy, such as low vitamin D status [25] or hypothyroidism [26]. Moreover, prematurely born infant girls were found to have higher serum LH, estradiol and inhibin B concentrations [27] and higher urinary FSH levels [28] than full-term controls, while infant girls born small for gestational age were characterized by higher serum FSH concentrations than girls born without growth restriction [29]. Although prematurity and abnormal birth size are frequent adverse neonatal outcomes of gestational diabetes mellitus [3,4], no previous research has assessed the association between hyperglycemia during pregnancy and minipuberty in the offspring. Thus, the aim of this study was to investigate whether maternal gestational diabetes mellitus affects hypothalamic–pituitary–ovarian axis activity and dimensions of female sexual organs (the ovaries, uterus and breasts) in the first 18 months of life.

## 2. Results

The study was completed by 82 patients (91.1%) whose results were statistically analyzed. Post-hoc power analysis showed that the study was sufficiently powered to detect meaningful differences. The required number of urine and saliva samples was not obtained from four patients (one assigned to group 1, two assigned to group 2, and one assigned to group 3) who developed chronic or recurrent infectious disease. Two infants (one from group 1 and one from group 2) were withdrawn because of severe gastroesophageal reflux and atopic dermatitis requiring therapy. One infant (allocated to group 1) prematurely ceased participation in the study because her mother was diagnosed with Graves’ disease, and the in-depth interview suggested that the disease might have developed at least several months earlier. Lastly, the parents of one infant (assigned to group 3) withdrew consent owing to personal reasons. 

At study entry, body weight and weight-for-length percentile were higher in group 2 than in the remaining two groups but did not differ between groups 1 and 3. There were no differences between the groups of infants in gestational age of delivery, birth order, length, head circumference, breastfeeding and total daily (with food and supplements) vitamin D intake (Table 1).

There were no between-group differences in age, education, occupational activity, type of work, smoking, BMI, blood pressure (both systolic and diastolic blood pressure) or mean daily vitamin D intake during pregnancy (with food and supplements) in mothers of infants who completed the study. Mean fasting glucose levels during pregnancy were higher in mothers of infants assigned to group 2 than in mothers of infants assigned to the remaining groups, but did not differ between groups 1 and 3 (Table 2). Similar proportions of women with gestational diabetes mellitus complied with dietary recommendations (group 2: 81%, group 3: 86%) and were treated with insulin (group 2: 19%, group 3: 21%). Both groups did not differ in cumulative insulin dose (1628 ± 528 units vs. 1820 ± 584 units, *p* = 0.5850). Compared to group 3, mothers of girls assigned to group 2 had during pregnancy higher mean levels of 1 h postprandial glucose (162 ± 15 mg/dL vs. 128 ± 10 mg/dL, *p* < 0.0001), 2 h postprandial glucose (135 ± 12 mg/dL vs. 104 ± 9 mg/dL, *p* < 0.0001) and HbA_1c_ (6.3 ± 0.2 mg/dL vs. 5.3 ± 0.2 mg/dL, *p* < 0.0001).

**Figure 1:**
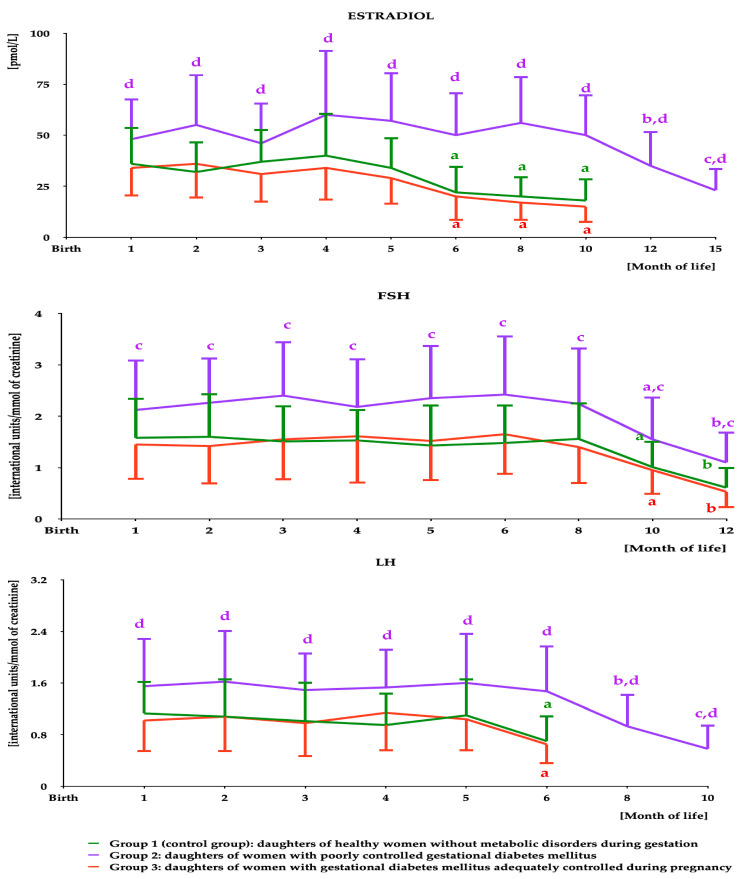
Salivary estradiol and urinary gonadotropin levels in infant girls participating in the study. The data are shown as the mean and standard deviation. Estradiol: undetectable at month 18 in group 1 and from month 12 to month 18 in the remaining groups. In statistical comparisons, LOD value was assigned for levels in groups 1 and 3 at months 12 and 15. ^a^*p* < 0.05 vs. levels during the first 5 months of life in the same study group; ^b^*p* < 0.05 vs. levels during the first 10 months of life in the same study group; ^c^*p* < 0.05 vs. levels during the first 12 months of life in the same study group; ^d^*p* < 0.05 vs. levels in groups 1 and 3 at the same time point. FSH: undetectable from month 15 to month 18. ^a^*p* < 0.05 vs. levels during the first 8 months of life in the same study group; ^b^*p* < 0.05 vs. levels during the first 10 months of life in the same study group; ^c^*p* < 0.05 vs. levels in groups 1 and 3 at the same time point. LH: undetectable from month 12 to month 18 in group 1, and from month 8 to month 18 in the remaining groups. In statistical comparisons, LOD value was assigned for levels in groups 1 and 3 at months 8 and 10. ^a^*p* < 0.05 vs. levels during the first 5 months of life in the same study group; ^b^*p* < 0.05 vs. levels during the first 6 months of life in the same study group; ^c^*p* < 0.05 vs. levels during the first 8 months of life in the same study group; ^d^*p* < 0.05 vs. levels in groups 1 and 3 at the same time point. Abbreviations: FSH—follicle-stimulating hormone; LH—luteinizing hormone; LOD—limit of detection.

Estradiol was detectable in saliva during the first 15 months of life in group 2 and during the first 10 months of life in the remaining groups. Significant decreases in estradiol levels were observed after month 10 in group 2 and after month 5 in groups 1 and 3. During the first 15 months of life, estradiol levels were higher in group 1 than in the remaining groups but did not differ between groups 1 and 3 (Figure 1 and Table S1).

In all study groups, progesterone was detectable in saliva during the first 12 months of life, and its concentrations were stable over the entire detection period. During the whole period of detection, progesterone concentration was lower in group 2 than in the remaining groups but did not differ between groups 1 and 3 (Table 3). 

Testosterone and DHEA-S were detectable in saliva during the first 5 and 12 months of life, respectively. There were no between-group differences in levels of both hormones (Table 4 and Table 5).

FSH was detectable in urine for the first 12 months of life. Stable hormone concentrations were observed from month 1 to month 8 and decreased thereafter. During the whole period of detection, FSH concentrations were higher in group 2 than in the remaining groups but did not differ between groups 1 and 3 (Figure 1 and Table S2).

LH was detectable in urine during the first 10 months of life in group 2 and during the first 6 months of life in groups 1 and 3. LH concentration was stable between months 1 and 6 in group 2, and between months 1 and 5 in the remaining two groups, and decreased thereafter. During the whole period of detection, urinary LH concentrations were higher in group 2 than in the remaining groups but did not differ between groups 1 and 3 (Figure 1 and Table S3).

In group 1, ovarian volume increased from month 1 to month 2, remained stable between months 2 and 10, and decreased thereafter. In group 2, ovarian volume remained stable for the first 12 months of life and decreased thereafter. In group 3, ovarian volume increased from month 1 to month 2, did not change between months 2 and 12, and was smaller at months 15 and 18. Over the entire study period, ovarian volume was greater in group 1 than in the remaining groups but did not differ between groups 2 and 3 (Table 6).

In group 2, uterine length remained stable over the entire study period. There was a decrease in uterine length between month 1 and month 2 in groups 1 and 3, with no changes in this parameter thereafter. From month 2 to month 18, the uterus was longer in group 2 than in the remaining groups, but the length did not differ between groups 1 and 3 (Table 7).

In group 2, breast size did not change throughout the study. In the remaining groups, there was a decrease in breast size between month 1 and month 2, with no changes in this diameter thereafter. From month 2 to month 18, breast diameter was larger in group 2 than in the remaining groups, but did not differ between groups 1 and 3 (Table 8).

Table 9 presents correlations between the measured variables. In all study groups, over the entire observation period, there were positive correlations between salivary estradiol concentration and urinary FSH concentration, between salivary estradiol concentration and urinary LH concentration, between ovarian volume and urinary gonadotropin levels, between uterine length and salivary estradiol concentration, between breast size and levels of estradiol and FSH. In group 2, mean maternal HbA_1c_ concentration positively correlated with FSH, LH and estradiol concentrations, and inversely correlated with progesterone concentrations. Other correlations did not reach statistical significance.

## 3. Discussion

This study shows for the first time that poorly controlled gestational diabetes of mothers results in an increased activity of the reproductive axis of their female offspring. Higher concentrations of gonadotropins and estradiol, as well as the presence of positive correlations between gonadotropins and estrogens and between gonadotropins and ovarian volume, indicate that increased estradiol production is associated with the impact of this disease at the hypothalamic and/or pituitary level. In addition to higher concentrations of gonadotropins and estradiol, the course of female minipuberty in this group was also characterized by a longer detection period for LH in urine and estradiol in saliva. The association between poor glycemic control during pregnancy and the altered course of minipuberty is supported by positive correlations between concentrations of FSH, LH and estradiol and levels of HbA_1c_. Our findings cannot be explained by the impact of differences in maternal content of adipose tissue (obese and overweight women are more prone to gestational diabetes mellitus [30]), because the study groups were matched for maternal BMI. From a theoretical point of view, the observed differences might have been associated with differences in body weight in the infants themselves. However, hormone levels and organ size in our study did not correlate with body weight and the weight-for-length percentile. Moreover, reproductive hormones in infant girls with Prader–Willi syndrome, a core symptom of which is obesity [31], did not differ from levels in prepubertal girls [32]. Owing to the strict inclusion criteria, differences in the course of minipuberty cannot be associated with differences in the gestational age of delivery or concurrent disorders. Lastly, increased activity of the reproductive axis cannot be explained by treatment (non-pharmacological and/or pharmacological). Similar proportions of mothers with gestational diabetes mellitus complied with lifestyle recommendations, and there were no statistically significant differences between women with poor and adequate disease control in insulin treatment. Moreover, although low vitamin D status during pregnancy was found to impact the course of minipuberty in the offspring [25], the study groups did not differ in total daily vitamin D intake, which was within the recommended range, ensuring normal vitamin D homeostasis [33]. It should be underlined that gestational diabetes mellitus is usually diagnosed in the second half of pregnancy, affects women with normal or only slightly impaired glucose tolerance before pregnancy, hyperglycemia is usually less expressed than in other forms of diabetes, and is very rarely accompanied by chronic micro- and macrovascular complications [34]. Thus, the course of female minipuberty in the descendants of mothers with poorly controlled type 1 and type 2 diabetes may be even more disturbed than in the analyzed population of infants. In line with previous observations [25,26], the detection period for estradiol in saliva was longer than for testosterone, as well as that there were no evident peaks in estradiol levels. Higher salivary estradiol concentrations in the first 10 months of life (infants of mothers with poorly controlled gestational diabetes mellitus) and in the first 5 months of life (the remaining infants) than afterward are in line with more pronounced ovarian production of this hormone during the first half of the first year of life than in the later periods of minipuberty, contrasting with minipubertal adrenal production that does not significantly change with age and is similar in both sexes [35]. The findings that salivary estradiol concentration correlated with the changes in ovarian volume and detection periods for this hormone were longer than for testosterone, as well as that DHEA-S concentrations remained stable throughout the study, indicate that estradiol in infant girls originates mainly from ovarian follicles. Although individual concentrations of estradiol (and possibly also of gonadotropins and progesterone) also fluctuate in this age group, reflecting the maturation and atrophy of ovarian follicles [28,36], the average concentrations may remain stable because folliculogenesis and follicular atrophy in various infants were temporarily uncoordinated. Considering no between-group differences in DHEA-S concentrations, adrenal androgen production does not seem to explain increased salivary estradiol in infants born to mothers with poorly controlled diabetes mellitus. Higher and observed for a longer period of time estradiol concentrations in the offspring of women with poorly controlled gestational diabetes may be partially associated with the impact on aromatase activity in ovarian granulomatosa cells and maybe also in extraovarian tissues. Although there are no data for fetuses and infants, reduced insulin sensitivity, characterizing gestational diabetes mellitus [34], stimulates the activity of this enzyme in adult female animals and humans [37,38].

The impact of gestational diabetes mellitus on female minipuberty was only partially similar to that of low vitamin D intake [25]. An important difference was low progesterone concentrations in the first year of life in daughters of mothers with poorly controlled gestational diabetes mellitus, contrasting with the unchanged concentrations of this hormone in infant girls born to vitamin D-deficient mothers. In turn, non-substituted or inadequately substituted hypothyroidism during pregnancy was associated with low gonadotropin and estradiol levels in infant girls and with a shorter period of their detection [26]. Although there are no other dedicated studies, these findings suggest that the association between female minipuberty and chronic disorders in pregnant women is disease-specific and does not seem to reflect a non-specific response of the reproductive axis to various pathologies during fetal life. Remarkable similarities between the course of minipuberty and puberty [14,15,16,17] in female descendants of women with gestational diabetes mellitus may suggest that the changes in the earliest phase of activation of the hypothalamic–pituitary–gonadal axis may beget the changes in the later phases. Thus, fetal exposure to hyperglycemia may theoretically result in overactivation of the reproductive axis already during the first phase. However, considering that the intrauterine phase takes place between gestational weeks 10 and 24 [7], while gestational diabetes is rather a complication of the second half of pregnancy [34], this explanation is less convincing. A more probable explanation is that chronic changes in the intrauterine environment have an impact on fetal programming [39]. In line with this explanation, hyperglycemia-induced epigenetic modifications of the fetal genome are implicated in the development of most adverse effects in the offspring of mothers with gestational diabetes mellitus [40], and they may also affect the course of the second phase of reproductive axis activation.

It should be underlined that there was no difference in hormone levels (and organ sizes) between infant girls born to women with gestational diabetes adequately controlled during pregnancy and daughters born to women with normal glucose homeostasis. This finding, which is in line with previous observations concerning descendants of women with gestational hypothyroidism [26,41], allows us to draw two conclusions. Firstly, effective treatment of hyperglycemia during pregnancy is likely associated with normal female minipuberty. Secondly, the study’s results underpin the rationale of recommended glycemic targets in pregnancy [42] and suggest avoiding HbA_1c_ exceeding 6.0% in women with GDM.

Another important finding resulting from our study is the significant differences in the size of sexual organs. Daughters born to women with poorly controlled gestational diabetes mellitus were characterized by greater dimensions of the ovaries, which are the main target for gonadotropins, but also by greater diameters of the uterus and breasts. Between-group differences in the size of the target organs were at least partially secondary to various degrees of activation of the reproductive axis. In line with this explanation, the size of the uterus and breasts correlated with salivary estradiol concentration and breast diameter, additionally, with FSH concentration, both of which were higher in the offspring of women with poorly controlled diabetes mellitus than in the remaining groups of infants. These markedly higher levels in the first month of life explain the lack of an initial decrease in the size of both organs between the first and second postnatal month in daughters born to mothers with inadequate glycemic control during pregnancy, though such a decrease was observed in daughters of women achieving the recommended levels of HbA_1c_. A similar decrease in uterine length and breast size was previously reported by another research group between day 7 and month 2 (breast size) and between day 7 and month 3 (uterine length) in full-term infant daughters of healthy, non-smoking women and was explained by the disappearance of maternal estrogens [36]. Thus, we cannot completely rule out a very early reduction in the size of these organs before the first study visit also in daughters of mothers with poorly controlled gestational diabetes mellitus. Between-group differences in uterine length and breast diameter were observed over the entire observation period, even at the last visit. It is possible that these differences may persist throughout the period of physiological quiescence of the hypothalamic–pituitary–ovarian axis until the beginning of puberty and may theoretically result in differences in the final (postpubertal) size of both organs, which should be verified in future research. Interestingly, although there are no data regarding disturbances of glucose homeostasis, girls with idiopathic central precocious puberty were characterized by a greater uterine volume if they were vitamin D-deficient, and this volume correlated with the severity of deficiency [43].

Another novel finding is low progesterone concentration in the offspring of mothers with poorly controlled gestational diabetes mellitus. The association with unsatisfactory disease control is backed up by negative correlations between this hormone and HbA_1c_. Because of the lack of correlation with ovarian volume and with levels of the remaining hormones, it is uncertain whether abnormally low progesterone concentration in this group of infants reflected reduced production by the ovaries or was a consequence of its decreased synthesis by the adrenal cortex. Moreover, because of the unproven role of progesterone in infancy, the significance of this finding is far from clear. However, at least theoretically, low progesterone concentration during minipuberty may be unfavorable because of the postulated role of this hormone in protecting the developing brain [44]. In adult women, progesterone is a negative feedback regulator of gonadotropin-releasing hormone pulse frequency, slowing LH pulse frequency and favoring FSH secretion [45]. Minipubertal progesterone production may theoretically explain higher levels of FSH than LH in saliva in the whole investigated population but does not explain why FSH predominance was also observed in the group with the lowest progesterone levels. Moreover, although progesterone opposes the regulatory actions of estrogen by the down-regulation of estrogen receptors in adults [46], our findings do not support a similar mechanism in female infants because there were no between-group differences in the strength of correlations between salivary estradiol levels and uterine length and breast diameter. Thus, the role of low progesterone in daughters of mothers with poorly controlled gestational diabetes mellitus requires further studies.

We can only speculate about the long-term consequences of altered minipuberty for reproductive health. Firstly, overactivity of the reproductive axis during minipuberty with subsequent between-group differences in dimensions of the assessed sexual organs may have an impact on the course of puberty, possibly explaining its earlier onset and faster progression in daughters of women with gestational diabetes mellitus, reported in previous studies [14,15,16,17,18]. Secondly, altered minipuberty may have an adverse effect on fertility. Although no dedicated human data are currently available, experiments in mouse models showed that adult female offspring of dams exposed in utero to gestational diabetes mellitus are subfertile, ovulate fewer oocytes, are characterized by a decreased number of preantral and antral follicles, and have a smaller litter size compared to offspring from normal pregnancies [47]. Lastly, increased follicular atresia following prenatal exposure to gestational diabetes mellitus [47] suggests that female offspring of women with this disorder may be at higher risk of premature ovarian failure.

There are some study limitations that merit consideration. Although the study was powered and the population exceeded the required number of individuals, our sample size was relatively small. The study excluded the offspring of women with type 1 and type 2 diabetes. Thus, the obtained results do not allow us to draw firm conclusions about minipuberty in the offspring of women with preexisting diabetes. The inclusion criteria allow us only to speculate about potential molecular explanations for our findings. Because almost all girls started participation in the study at the age of between 16 and 30 days, it is difficult to conclude about the impact of gestational diabetes mellitus on the earliest phase of female minipuberty (during the first two weeks of life). Owing to the study design, the findings could have been influenced by bias associated with the selection of the study population and/or with confounding variables. Because of the lack of specificity of the antibodies, immunoassays (used in our study) may overestimate steroid concentrations due to cross-reactivity with molecules similar to the analyte [48]. Lastly, precautions during study design and data analysis only limited, but did not completely eliminate, the regression toward the mean [49].

**Figure 2:**
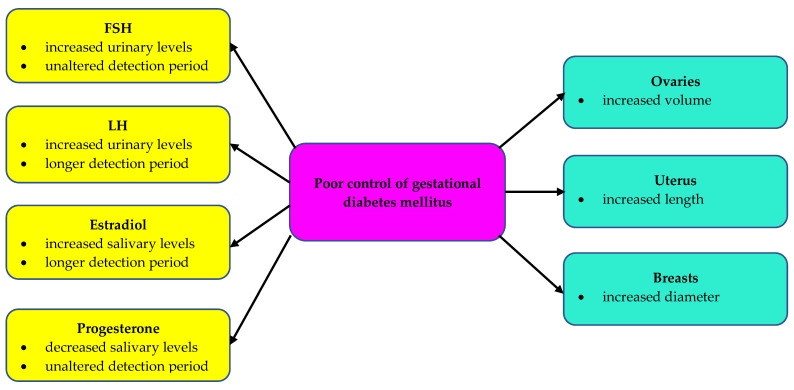
Reproductive hormones and sexual organs in infant girls born to mothers with poorly-controlled gestational diabetes mellitus.

In summary, infant girls born to women with poorly controlled gestational diabetes mellitus are characterized by altered levels of reproductive hormones, differences in their detection periods and larger dimensions of sexual organs (Figure 2). These changes depend on the degree of glycemic control in mothers during pregnancy and are absent in the offspring of women with the adequately controlled disease. 

## 4. Materials and Methods

This single-center, prospective, matched, outpatient cohort study was conducted between June 2022 and August 2024. Because the study did not prospectively assign patients to a health-related intervention, trial registration was not required. The study protocol was approved by the institutional review board (the Bioethical Committee of the Medical University of Silesia [approval number: PCN/CBN/0052/KB1/17/22, date: 12 April 2022]) before the research began, and the study was completed in accordance with the Helsinki Declaration as revised in 2013. A parent and/or legal guardian of each infant gave written informed consent during the enrollment process after being informed about the study details.

### 4.1. Participants

The study population included 90 apparently healthy girls under 30 days of age, initially supervised by neonatologists and pediatricians cooperating with our research group. Based on routine screening of mothers for gestational diabetes mellitus, the infants were allocated into one of three groups, each including 30 girls: daughters of healthy women without metabolic disorders during gestation (group 1), daughters of women with poorly controlled gestational diabetes mellitus (group 2), and female descendants of women with gestational diabetes mellitus adequately controlled during pregnancy (group 3). Gestational diabetes mellitus was diagnosed based on the World Health Organization 2013 recommendations if between weeks 24 and 28 of pregnancy, or at any other time in pregnancy at least one of the following criteria was met: fasting plasma glucose 92–125 mg/dL (5.1–6.9 mmol/L); one-hour plasma glucose following a 75 g oral glucose load greater or equal to 180 mg/dL (10.0 mmol/L); and two-hour plasma glucose following a 75 g oral glucose load between 153 and 199 mg/dL (between 8.5 and 11.0 mmol/L) [1]. Mothers were considered healthy if fasting or post-challenge glucose levels were below these threshold values. If measured, glycated hemoglobin (HbA_1c_) had to be below 5.7% (39 mmol/mol). GDM was considered adequately controlled if the following criteria were met: mean fasting plasma glucose below 95 mg/dL (5.3 mmol/L), mean 1 h postprandial glucose below 140 mg/dL (7.8 mmol/L), mean 2 h postprandial glucose below 120 mg/dL (6.7 mmol/L), and mean HbA_1c_ below 6.0% (42 mmol/mol). If mean values of glucose and HbA_1c_ were above these threshold levels, the disease was regarded as poorly controlled. 

An *a priori* sample size calculation showed that at least 24 infants per group were needed to detect a 20% between-group difference in estradiol concentration (the primary endpoint) with a power of 0.8 and α of 5%. Considering possible dropouts, the size of each group was increased by 25%. The study population was selected from a larger cohort of infant girls meeting all inclusion criteria (n = 164) in order to obtain the study groups matched for maternal age, maternal BMI, gestational age of delivery and birth order. The algorithm used in the matching procedure was based on the minimum Euclidean distance rule [50]. All necessary data concerning pregnancy and delivery were retrieved from the medical records of mothers and neonates. To minimize seasonal confounds and fluctuations in the investigated hormones, the study included similar numbers of infants born between June and August (eight in each group), between September and November (seven in each group), between December and February (seven in group 2, eight in groups 1 and 3), and between March and May (eight in group 2, seven in groups 1 and 3).

Infants were not considered for enrollment if mothers had preexisting type 1 or type 2 diabetes, overt diabetes in pregnancy, preexisting prediabetes, or glucose or HbA_1c_ levels were inconclusive. Infants were also excluded in case of chronic disorders in mothers, maternal complications requiring urgent hospitalization during pregnancy, any medical treatment exceeding 10 days during pregnancy or lactation (except for insulin and vitamin/micronutrient supplements for pregnant and breastfeeding women) and maternal addiction to drugs or alcohol. Lastly, the exclusion criteria included genetic or inherited syndromes, major congenital anomalies, congenital infections, delivery before week 36 of gestation, a history of birth asphyxia, metabolic disorders, other chronic diseases and chronic pharmacotherapy (except for vitamin D supplements).

### 4.2. Study Design

**Figure 3:**
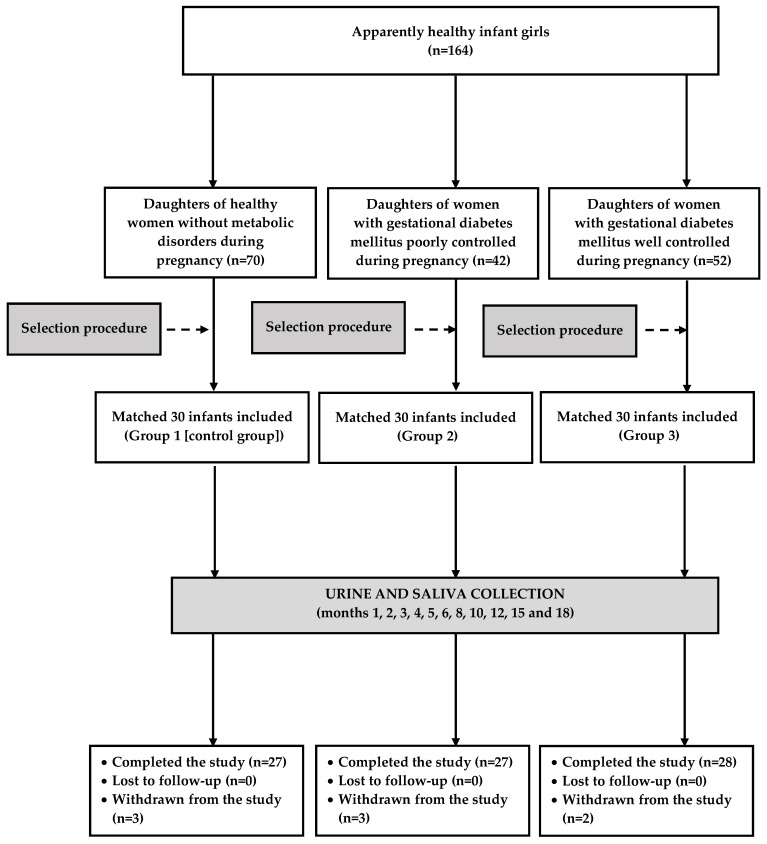
The flow of the participants through the study.

Figure 3 illustrates the flow of patients through the study (from screening to the analysis). The infant girls were followed up from postnatal month 1 to postnatal month 18: once a month for the first six months of life, every other month for the following six months, and once every three months thereafter. The parents or guardians were asked to describe the infant’s health condition and development since the last follow-up visit and about medication usage. Moreover, the members of the research team performed a thorough physical examination and analyzed the results of laboratory and imaging tests (if they were performed). Anthropometric measurements were recorded, and the biological material (urine and saliva) was collected only if the child was considered healthy and did not receive any treatment during the last 10 days (except for mandatory vaccinations). The infants were considered eligible for the final analysis only if these procedures were performed eight times or more often (at least fivefold between month 1 and month 6, at least twice between month 6 and month 12, and at least once between month 12 and month 18).

Anthropometric measurements of supine length, weight and head circumference were performed at each study visit. Length was measured from the crown of the head to the foot’s heel, while both were appropriately attached to the infantometer (Seca, Hamburg, Germany). Head circumference was measured from the occiput to the supraorbital ridges using a flexible measuring tape. Weight was measured using a digital infant scale (Seca 834, Hamburg, Germany) with clothing and diapers removed. Measurements were recorded to the nearest 1 mm (length and head circumference) or 10 g (weight). Weight-for-length was calculated by dividing the infant’s weight by length and then converted to percentiles using the World Health Organization growth charts. BMI (in mothers) was calculated as weight in kilograms divided by squared length in meters. BMI was calculated as weight divided by height squared and expressed in kg/m^2^. 

Ovarian volume and uterine length were measured using a high-resolution, small-part transducer with a frequency of 5–12 MHz (Esaote MyLab Six, Genova, Italy). Three independent measurements were always taken, and the obtained results were averaged. Transabdominal sonographic examinations were performed and interpreted by the investigators. The examination was performed with the infant in a supine position. The investigator waited for the bladder to be full so as to obtain the best possible image (the bladder served as an acoustic window). Ovarian volume was computed in cubic centimeters according to the formula for prolate ellipse: volume = longitudinal diameter (in cm) × transverse diameter (in cm) × anteroposterior diameter (in cm) × 0.52. If both the left and right ovary were visualized, then the average volume of the two was used. If only one ovary could be evaluated, this ovarian volume was used. Uterine length was measured from the fundus to the external opening at the maximum length of the midsagittal section. The presence of breast tissue was determined by palpation, and its size was measured to the nearest millimeter using a caliper (Insize Europe, Derio, Spain). Diameters below 3 mm were considered unmeasurable (equivalent to the nipple diameter), and their size was assumed at 1 mm [51]. The sum of the diameters of mammary glands was then divided by two to calculate the mean diameter.

### 4.3. Laboratory Assays

To avoid interference from the circadian cycle [52], urine and saliva samples were collected between 8 and 9.30 a.m. in a quiet, air-conditioned room. Urine samples were collected from all infants using adhesive urine bags (Medicavera, Szczecin, Poland). After cleansing and drying the skin around the genital area and removing protective backing from the adhesive patch, the bag was attached to the patient’s perineum and left in place for at least one hour unless there was evidence of leakage, stool contamination, or the bag separated from the skin. The bag was then emptied into the container by cutting the projection at the bottom corner. Saliva was aspirated by gentle suction from the floor of the infant’s mouth using a 2 mL sterile plastic syringe (Polfa Lublin, Lublin, Poland). The whole procedure lasted only 30–50 s and was well tolerated by the infants. In order to minimize the risk of contamination and the possible impact of feeding on the secretion and/or metabolism of the measured hormones, they were not fed for at least one hour prior to the collection procedure. The saliva was centrifuged immediately after the collection for 15 min at 40,000× *g* to remove the epithelial cells and suspended matter, and the supernatants were aliquoted in polypropylene tubes. Urine and saliva samples were then stored at −20 °C until analysis.

All measurements were performed on undiluted samples in duplicate to confirm reproducibility. Urinary concentrations of FSH and LH were assayed by chemiluminescent immunometric assay, and the obtained results were adjusted for creatinine (assayed using a routine laboratory technique) to account for urine dilution as described previously [53]. Levels of estradiol, progesterone, testosterone and dehydroepiandrosterone sulfate (DHEA-S) in saliva were assessed using an enzyme-linked immunosorbent assay [41,54]. Kits were purchased from Salimetrics (Carlsbad, CA, USA) [estradiol and DHEA-S], BioVendor R&D (Brno, Czech Republic) [progesterone], DiaMetra (Perugia, Italy) [testosterone] and Abbott Laboratories (Green Oaks, IL, USA) [FSH and LH]. Catalog numbers were as follows: 1-3702 (estradiol), RTC016R (progesterone), DKO021 (testosterone), 1-1252-5 (DHEA-S), 7K75 (FSH) and 2P40 (LH). The inter-assay coefficients of variation were 6.0% for estradiol, 6.5% for progesterone, 7.2% for testosterone, 7.0% for DHEA-S, 4.1% for FSH and 4.6% for LH. Limits of detection (LOD) were as follows: 4 pmol/L for estradiol, 10 pmol/L for testosterone, 100 nmol/L for DHEA-S, 16 pmol/L for progesterone and 0.1 U/L for FSH and LH.

### 4.4. Statistical Analysis

All quantitative data were logarithmically transformed to meet the model assumptions of normality and homoscedasticity. Comparisons between the groups at the same time point were performed using one-way analysis of variance followed by Bonferroni’s test. Within-group comparisons were made using repeated measures analysis of variance, followed by Tukey’s post hoc test for pairwise comparisons. If measurements were below LOD, the LOD value was assigned for statistical comparisons. Qualitative variables were compared using the chi-square test. Bivariate relationships were analyzed using Pearson’s r tests (for two parametric variables), phi coefficient (for one parametric and one categorical variable) and point-biserial (for two categorical variables). Statistical significance was defined as a *p*-value less than 0.05. All statistical calculations were carried out using the Statistica 12.5 PL software package (number: ZZS999000009912294-B, StatSoft Polska, Kraków, Poland).

## 5. Conclusions

If poorly controlled, gestational diabetes mellitus may be complicated by the abnormal course of minipuberty in female offspring, including increased and prolonged activation of the hypothalamic–pituitary–ovarian axis and lower salivary progesterone levels. Overactivation of the reproductive axis is likely to be responsible for greater dimensions of the ovaries, uterus and breasts, present for almost the whole period of infancy. These abnormalities may have an unfavorable impact on female reproductive health at later stages of life. Changes in hypothalamic–pituitary–ovarian axis activity during infancy seem to be determined by the severity of hyperglycemia during pregnancy. The course of minipuberty is unaffected if mothers achieve recommended glycemic goals. Because of novelty, potential clinical relevance and study limitations, the obtained results need to be confirmed in larger, prospective, controlled clinical studies. It would also be worthwhile to investigate the course of minipuberty in sons of mothers with gestational diabetes mellitus and in children of women with preexisting diabetes and to understand in-depth the molecular mechanisms underlying our findings.

## Figures and Tables

**Table 1:** Baseline characteristics of infant girls who completed the study.

Variable	Group 1	Group 2	Group 3
Number (n)	27	27	28
Gestational age of delivery (weeks)	40 ± 1	39 ± 2	40 ± 2
Birth order: first/second/third and subsequent (%)	48/41/11	52/41/7	50/43/7
Body length (cm)	54.2 ± 1.7	53.9 ± 1.7	54.1 ± 1.8
Head circumference (cm)	37.1 ± 0.8	37.4 ± 1.0	36.9 ± 0.9
Body weight (kg)	4.30 ± 0.52	4.61 ± 0.58^a^	4.26 ± 0.56
Weight-for-length percentile	52 ± 19	80 ± 10^a^	48 ± 21
Breastfeeding (%)	85	82	86
Total daily vitamin D intake (µg)	12.7 ± 1.6	12.9 ± 1.3	13.1 ± 1.4

Unless otherwise stated, the data are shown as the mean ± standard deviation. Group 1: daughters of healthy women without metabolic disorders during gestation (control group); group 2: daughters of women with poorly controlled gestational diabetes mellitus; group 3: daughters of women with gestational diabetes mellitus adequately controlled during pregnancy. ^a^*p* < 0.05 vs. respective value in groups 1 and 3.

**Table 2:** Characteristics of mothers of infants who completed the study.

Variable	Group 1	Group 2	Group 3
Number (n)	27	27	28
Age (years)	34 ± 7	35 ± 8	36 ± 8
University/secondary/primary or vocational education (%)	52/41/7	56/37/7	50/39/11
Employment rate/white-collar/pink-collar/blue-collar workers (%)	89/48/30/11	93/52/30/11	93/50/32/7
Smokers (%)	37	37	39
BMI (kg/m^2^)	24.2 ± 4.2	25.3 ± 4.4	25.1 ± 4.0
Systolic blood pressure (mmHg)	117 ± 20	123 ± 18	119 ± 15
Diastolic blood pressure (mmHg)	79 ± 7	82 ± 8	80 ± 7
Mean daily vitamin D intake during pregnancy (µg)	48.6 ± 15.8	42.5 ± 12.8	44.1 ± 12.9
Mean fasting glucose during pregnancy (mg/dL)	78 ± 9	100 ± 10^a^	79 ± 8

Unless otherwise stated, the data are shown as the mean ± standard deviation. Group 1: healthy women without metabolic disorders during gestation (control group); group 2: women with poorly controlled gestational diabetes mellitus; group 3: women with gestational diabetes mellitus adequately controlled during pregnancy. Abbreviations: BMI—body mass index; ^a^*p* < 0.05 vs. respective value in groups 1 and 3.

**Table 3:** Progesterone concentration in saliva of infant girls participating in the study.

Age [month]	Group 1	Group 2	Group 3
1	80 ± 52	50 ± 30^a^	78 ± 40
2	72 ± 46	48 ± 30^a^	80 ± 35
3	69 ± 40	39 ± 22^a^	65 ± 42
4	82 ± 50	47 ± 28^a^	73 ± 39
5	74 ± 41	43 ± 20^a^	84 ± 42
6	80 ± 48	52 ± 30^a^	82 ± 36
8	67 ± 39	44 ± 29^a^	76 ± 39
10	78 ± 40	40 ± 23^a^	78 ± 42
12	64 ± 38	38 ± 25^a^	68 ± 38

The data are expressed in pmol/L and shown as the mean ± standard deviation. In all groups, progesterone was undetectable from month 12 to month 18. Group 1: daughters of healthy women without metabolic disorders during gestation (control group); group 2: daughters of women with poorly controlled gestational diabetes mellitus; group 3: daughters of women with gestational diabetes mellitus adequately controlled during pregnancy. ^a^*p* < 0.05 vs. levels in groups 1 and 3 at the same time point.

**Table 4:** Testosterone concentration in saliva of infant girls participating in the study.

Age [month]	Group 1	Group 2	Group 3
1	56 ± 32	50 ± 25	47 ± 21
2	50 ± 27	46 ± 20	51 ± 24
3	46 ± 26	52 ± 32	43 ± 26
4	42 ± 21	46 ± 25	50 ± 30
5	44 ± 24	40 ± 20	42 ± 17

The data are expressed in pmol/L and shown as the mean ± standard deviation. In all groups, testosterone was undetectable from month 6 to month 18. Group 1: daughters of healthy women without metabolic disorders during gestation (control group); group 2: daughters of women with poorly controlled gestational diabetes mellitus; group 3: daughters of women with gestational diabetes mellitus adequately controlled during pregnancy.

**Table 5:** DHEA-S concentration in saliva of infant girls participating in the study.

Age [month]	Group 1	Group 2	Group 3
1	168 ± 86	155 ± 85	165 ± 95
2	157 ± 90	162 ± 90	173 ± 108
3	175 ± 110	146 ± 79	140 ± 69
4	163 ± 82	128 ± 82	167 ± 92
5	149 ± 80	134 ± 75	170 ± 105
6	159 ± 78	158 ± 80	153 ± 88
8	171 ± 100	164 ± 89	145 ± 84
10	148 ± 68	153 ± 78	140 ± 70
12	122 ± 69	130 ± 80	120 ± 75

The data are expressed in nmol/L and shown as the mean ± standard deviation. In all groups, DHEA-S was undetectable from month 15 to month 18. Group 1: daughters of healthy women without metabolic disorders during gestation (control group); group 2: daughters of women with poorly controlled gestational diabetes mellitus; group 3: daughters of women with gestational diabetes mellitus adequately controlled during pregnancy. Abbreviation: DHEA-S—dehydroepiandrosterone sulfate.

**Table 6:** Ovarian volume in infant girls participating in the study.

Age [month]	Group 1	Group 2	Group 3
1	0.78 ± 0.30	1.10 ± 0.41^d^	0.76 ± 0.24
2	0.97 ± 0.32^a^	1.30 ± 0.49^d^	0.95 ± 0.30^a^
3	1.08 ± 0.28^a^	1.27 ± 0.53^d^	0.98 ± 0.34^a^
4	1.12 ± 0.41^a^	1.32 ± 0.50^d^	1.00 ± 0.29^a^
5	1.03 ± 0.38^a^	1.24 ± 0.44^d^	1.10 ± 0.38^a^
6	1.08 ± 0.40^a^	1.29 ± 0.48^d^	1.05 ± 0.40^a^
8	1.10 ± 0.35^a^	1.34 ± 0.56^d^	1.13 ± 0.46^a^
10	1.03 ± 0.28^a^	1.26 ± 0.43^d^	1.04 ± 0.38^a^
12	0.82 ± 0.31^b^	1.31 ± 0.51^d^	0.96 ± 0.37^a^
15	0.70 ± 0.29^b^	0.88 ± 0.32^a,c,d^	0.67 ± 0.30^b^
18	0.68 ± 0.26^b^	0.86 ± 0.29^a,c,d^	0.69 ± 0.32^b^

The data are expressed in cm^3^ and shown as the mean ± standard deviation. Group 1: daughters of healthy women without metabolic disorders during gestation (control group); group 2: daughters of women with poorly controlled gestational diabetes mellitus; group 3: daughters of women with gestational diabetes mellitus adequately controlled during pregnancy. ^a^*p* < 0.05 vs. volume at month 1 in the same study group; ^b^*p* < 0.05 vs. volumes at months 2–10 in the same study group; ^c^*p* < 0.05 vs. volumes at months 2–12 in the same study group; ^d^*p* < 0.05 vs. levels in groups 1 and 3 at the same time point.

**Table 7:** Uterine length in infant girls participating in the study.

Age [month]	Group 1	Group 2	Group 3
1	35 ± 6	36 ± 6	35 ± 5
2	31 ± 5^a^	35 ± 6^b^	31 ± 6^a^
3	31 ± 6^a^	37 ± 5^b^	30 ± 6^a^
4	30 ± 4^a^	37 ± 6^b^	30 ± 6^a^
5	31 ± 5^a^	36 ± 4^b^	30 ± 5^a^
6	31 ± 5^a^	35 ± 6^b^	31 ± 5^a^
8	30 ± 5^a^	35 ± 5^b^	29 ± 5^a^
10	29 ± 5^a^	35 ± 5^b^	30 ± 5^a^
12	30 ± 5^a^	36 ± 5^b^	29 ± 5^a^
15	29 ± 5^a^	36 ± 6^b^	30 ± 5^a^
18	30 ± 6^a^	36 ± 4^b^	29 ± 5^a^

The data are expressed in mm and shown as the mean ± standard deviation. Group 1: daughters of healthy women without metabolic disorders during gestation (control group); group 2: daughters of women with poorly controlled gestational diabetes mellitus; group 3: daughters of women with gestational diabetes mellitus adequately controlled during pregnancy. ^a^*p* < 0.05 vs. length at month 1 in the same study group; ^b^*p* < 0.05 vs. length in groups 2 and 3 at the same time point.

**Table 8:** Breast diameter in infant girls participating in the study.

Age [month]	Group 1	Group 2	Group 3
1	13 ± 4	14 ± 4	13 ± 5
2	9 ± 5^a^	13 ± 5^b^	9 ± 4^a^
3	8 ± 3^a^	12 ± 4^b^	9 ± 5^a^
4	9 ± 4^a^	13 ± 4^b^	9 ± 5^a^
5	8 ± 5^a^	13 ± 5^b^	8 ± 4^a^
6	8 ± 4^a^	12 ± 5^b^	8 ± 4^a^
8	8 ± 3^a^	14 ± 5^b^	9 ± 4^a^
10	9 ± 4^a^	14 ± 5^b^	8 ± 4^a^
12	7 ± 3^a^	13 ± 4^b^	7 ± 3^a^
15	7 ± 4^a^	12 ± 5^b^	8 ± 5^a^
18	7 ± 3^a^	14 ± 5^b^	7 ± 4^a^

The data are expressed in mm and shown as the mean ± standard deviation. Group 1: daughters of healthy women without metabolic disorders during gestation (control group); group 2: daughters of women with poorly controlled gestational diabetes mellitus; group 3: daughters of women with gestational diabetes mellitus adequately controlled during pregnancy. ^a^*p* < 0.05 vs. diameter at month 1 in the same study group; ^b^*p* < 0.05 vs. diameter in groups 1 and 3 at the same time point.

**Table 9:** Correlations between the measured variables.

Correlated Variables		Group 1	Group 2	Group 3
Estradiol	FSH	0.46 [*p* = 0.0002]–0.67 [*p* < 0.0001]	0.40 [*p* = 0.0012]–0.56 [*p* < 0.0001]	0.44 [*p* = 0.0003]–0.68 [*p* < 0.0001]
Estradiol	LH	0.32 [*p* = 0.0426]–0.46 [*p* = 0.0002]	0.32 [*p* = 0.0308]–0.43 [*p* = 0.0004]	0.30 [*p* = 0.0412]–0.48 [*p* = 0.0001]
Ovarian volume	LH	0.34 [*p* = 0.0258]–0.42 [*p* = 0.0005]	0.29 [*p* = 0.0395]–0.41 [*p* = 0.0010]	0.32 [*p* = 0.0302]–0.44 [*p* = 0.0003]
Ovarian volume	FSH	0.25 [*p* = 0.0472]–0.39 [*p* = 0.0125]	0.24 [*p* = 0.0498]–0.36 [*p* = 0.0087]	0.25 [*p* = 0.0480]–0.38 [*p* = 0.0141]
Uterine length	Estradiol	0.30 [*p* = 0.0272]–0.42 [*p* = 0.0005]	0.26 [*p* = 0.0402]–0.40 [*p* = 0.0010]	0.28 [*p* = 0.0351]–0.43 [*p* = 0.0004]
Breast diameter	Estradiol	0.32 [*p* = 0.0298]–0.46 [*p* = 0.0006]	0.31 [*p* = 0.0328]–0.44 [*p* = 0.0003]	0.29 [*p* = 0.0459]–0.46 [*p* = 0.0002]
Breast diameter	FSH	0.34 [*p* = 0.0241]–0.46 [*p* = 0.0002]	0.34 [*p* = 0.0262]–0.46 [*p* = 0.0002]	0.32 [*p* = 0.0311]–0.47 [*p* = 0.0001]
Mean maternal HbA_1c_	FSH	not assessed	0.35 [*p* = 0.0230]–0.47 [*p* = 0.0001]	0.12 [*p* = 0.2983]–0.22 [*p* = 0. 0537]
Mean maternal HbA_1c_	LH	not assessed	0.29 [*p* = 0.0467]–0.43 [*p* = 0.0005]	0.15 [*p* = 0.1893]–0.21 [*p* = 0.0658]
Mean maternal HbA_1c_	Estradiol	not assessed	0.35 [*p* = 0.0224]–0.46 [*p* = 0.0002]	0.04 [*p* = 0.7885]–0.20 [*p* = 0.0843]
Mean maternal HbA_1c_	Progesterone	not assessed	−0.32 [*p* = 0.0346]–−0.44 [*p* = 0.0004]	−0.10 [*p* = 0.4060]–−0.22 [*p* = 0.0605]

The data represent the correlation coefficients (r values). Group 1: daughters of healthy women without metabolic disorders during gestation (control group); group 2: daughters of women with poorly controlled gestational diabetes mellitus; group 3: daughters of women with gestational diabetes mellitus adequately controlled during pregnancy. Abbreviations: FSH—follicle-stimulating hormone; HbA_1c_—glycated hemoglobin; LH—luteinizing hormone.

## Data Availability

The data that support the findings of this study are available from the corresponding author upon reasonable request.

## Supplementary Materials

The following supporting information can be downloaded at https://www.mdpi.com/article/10.3390/ijms252111766/s1.

## Abbreviations

BMI—body mass index; DHEA-S—dehydroepiandrosterone sulfate; FSH—follicle-stimulating hormone; HbA1c—glycated hemoglobin; LH—luteinizing hormone; LOD—limit of detection.
