# Supplementary material for: The Impact of Gestational Diabetes Mellitus on Minipuberty in Girls
Source: Int J Mol Sci. 2024 Nov 1;25(21):11766. doi: 10.3390/ijms252111766 (PMC11545881; doi:10.3390/ijms252111766)
Supplement: Supplementary file 1 [file ijms-25-11766-s001.zip › ijms-3258358-supplementary.pdf]

Supplementary Table S1. Estradiol concentration in saliva of infant girls participating in the study.

Age [month]	Group 1	Group 2	Group 3
1	36±18	48±20[d]	34±14
2	32±15	55±25[d]	36±17
3	37±16	46±20[d]	31±14
4	40±21	60±32[d]	34±16
5	34±15	57±24[d]	29±13
6	22±13[a]	50±21[d]	20±12[a]
8	20±10[a]	56±23[d]	17±9[a]
10	18±11[a]	50±20[d]	15±8[a]
12	Below LOD	35±17[b,d]	Below LOD
15	Below LOD	23±11[c,d]	Below LOD

The data are expressed in pmol/L and shown as the mean ± standard deviation. Estradiol was undetectable at month 18 in group 1, and from month 12 to month 18 in the remaining groups. In statistical comparisons, LOD value was assigned for estradiol in groups 1 and 3 at months 12 and 15. Group 1: daughters of healthy women without metabolic disorders during gestation (control group); group 2: daughters of women with poorly controlled gestational diabetes mellitus; group 3: daughters of women with gestational diabetes mellitus adequately controlled during pregnancy. [a]$p<0.05$ *vs*. levels during the first 5 months of life in the same study group; [b]$p<0.05$ *vs*. levels during the first 10 months of life in the same study group; [c]$p<0.05$ *vs*. levels during the first 12 months of life in the same study group; [d]$p<0.05$ *vs*. levels in groups 1 and 3 at the same time point. Abbreviation: LOD - limit of detection.

Supplementary Table S2. Urinary FSH concentration in infant girls participating in the study.

Age [month]	Group 1	Group 2	Group 3
1	1.51±0.78	2.12±0.98[c]	1.45±0.69
2	1.60±0.85	2.26±0.88[c]	1.42±0.75
3	1.51±0.70	2.40±1.06[c]	1.55±0.80
4	1.53±0.61	2.18±0.95[c]	1.61±0.92
5	1.43±0.80	2.35±1.04[c]	1.52±0.78
6	1.48±0.75	2.42±1.15[c]	1.65±0.79
8	1.56±0.71	2.24±1.10[c]	1.40±0.72
10	1.01±0.51[a]	1.55±0.83[a,c]	0.95±0.48[a]
12	0.61±0.40[b]	1.10±0.60[b,c]	0.53 ±0.32[b]

The data are expressed in international units per mmol of creatinine and shown as the mean ± standard deviation. FSH was undetectable from month 15 to month 18. Group 1: daughters of healthy women without metabolic disorders during gestation (control group); group 2: daughters of women with poorly controlled gestational diabetes mellitus; group 3: daughters of women with gestational diabetes mellitus adequately controlled during pregnancy. [a]$p<0.05$ *vs*. levels during the first 8 months of life in the same study group; [b]$p<0.05$ *vs*. levels during the first 10 months of life in the same study group; [c]$p<0.05$ *vs*. levels in groups 1 and 3 at the same time point. Abbreviation: FSH - follicle-stimulating hormone.

Supplementary Table S3. Urinary LH concentration in infant girls participating in the study.

Age [month]	Group 1	Group 2	Group 3
1	1.13±0.50	1.55±0.75[d]	1.02±0.49
2	1.08±0.59	1.62±0.80[d]	1.08±0.55
3	1.01±0.61	1.49±0.58[d]	0.98±0.53
4	0.95±0.50	1.53±0.60[d]	1.14±0.60
5	1.10±0.57	1.60±0.78[d]	1.04±0.50
6	0.70±0.40[a]	1.47±0.71[d]	0.65±0.31[a]
8	Below LOD	0.93±0.50[b,d]	Below LOD
10	Below LOD	0.58±0.38[c,d]	Below LOD

The data are expressed in international units per mmol of creatinine and shown as the mean ± standard deviation. LH was undetectable from month 12 to month 18 in group 1, and from month 8 to month 18 in the remaining groups. In statistical comparisons, LOD value was assigned for LH in groups 1 and 3 at months 8 and 10. Group 1: daughters of healthy women without metabolic disorders during gestation (control group); group 2: daughters of women with poorly controlled gestational diabetes mellitus; group 3: daughters of women with gestational diabetes mellitus adequately controlled during pregnancy. [a]$p<0.05$ *vs*. levels during the first 5 months of life in the same study group; [b]$p<0.05$ *vs*. levels during the first 6 months of life in the same study group; [c]$p<0.05$ *vs*. levels during the first 8 months of life in the same study group; [d]$p<0.05$ *vs*. levels in groups 1 and 3 at the same time point. Abbreviations: LH - luteinizing hormone; LOD - limit of detection.
